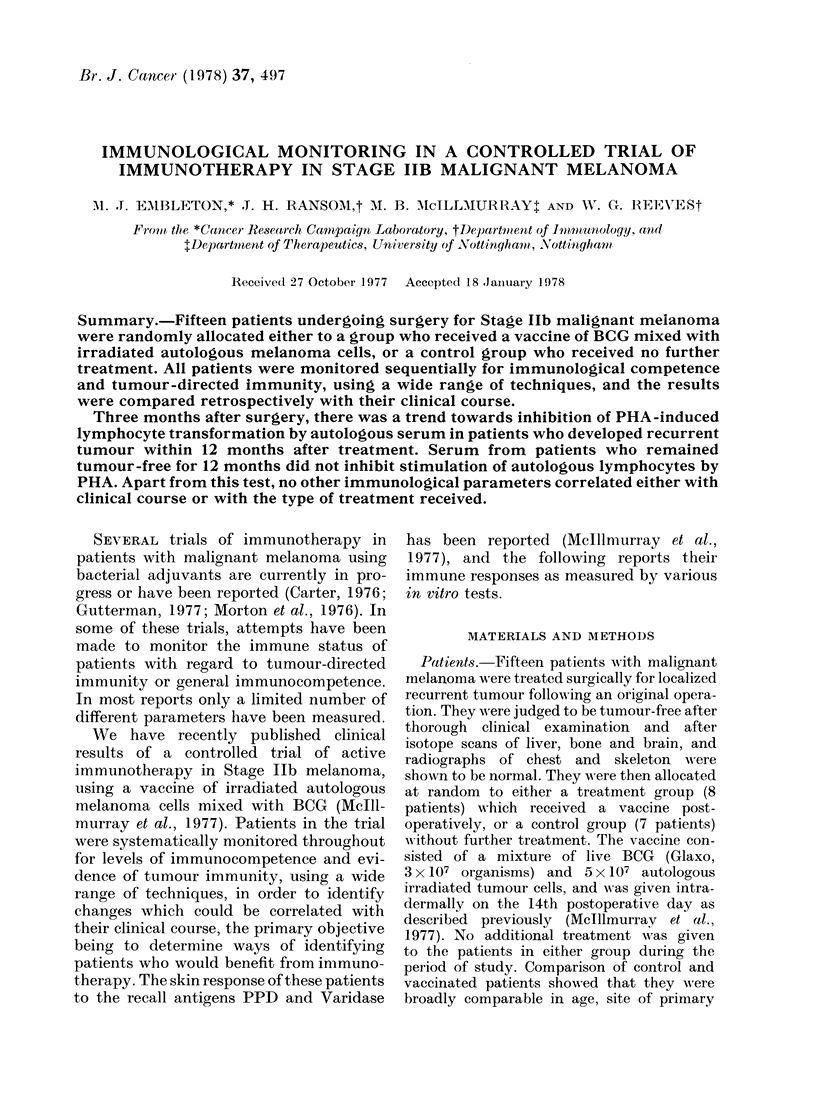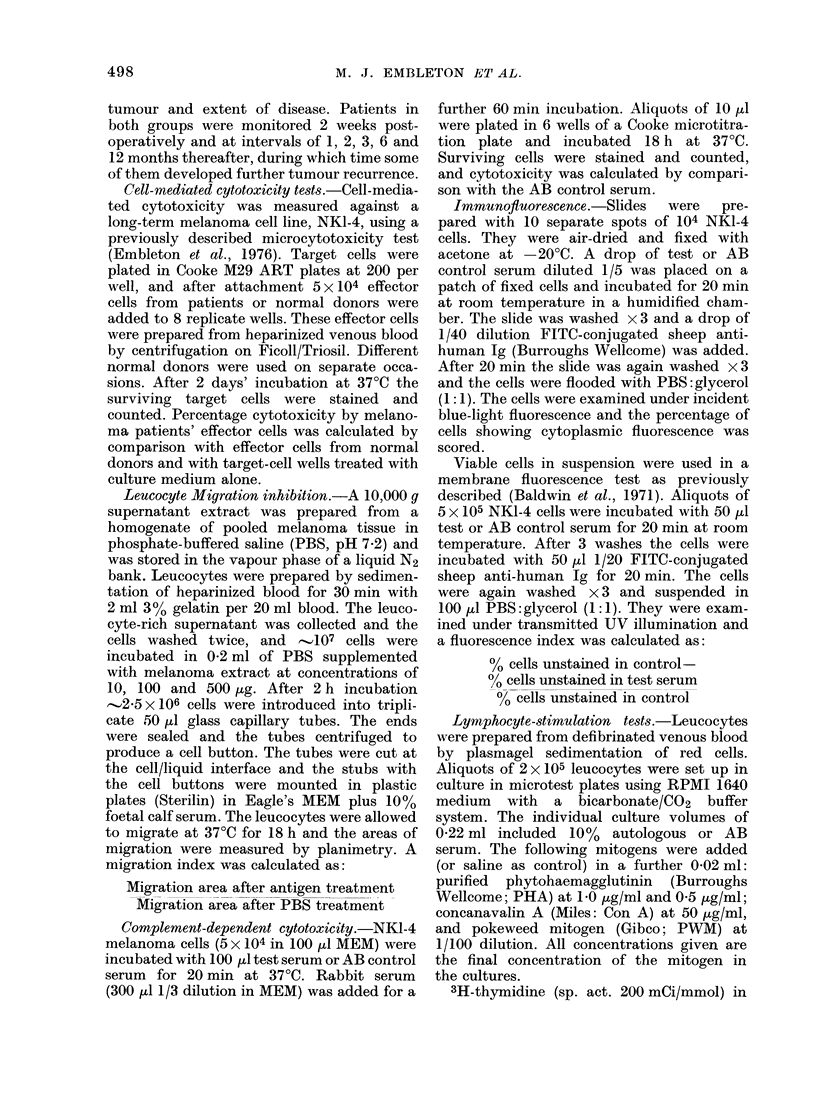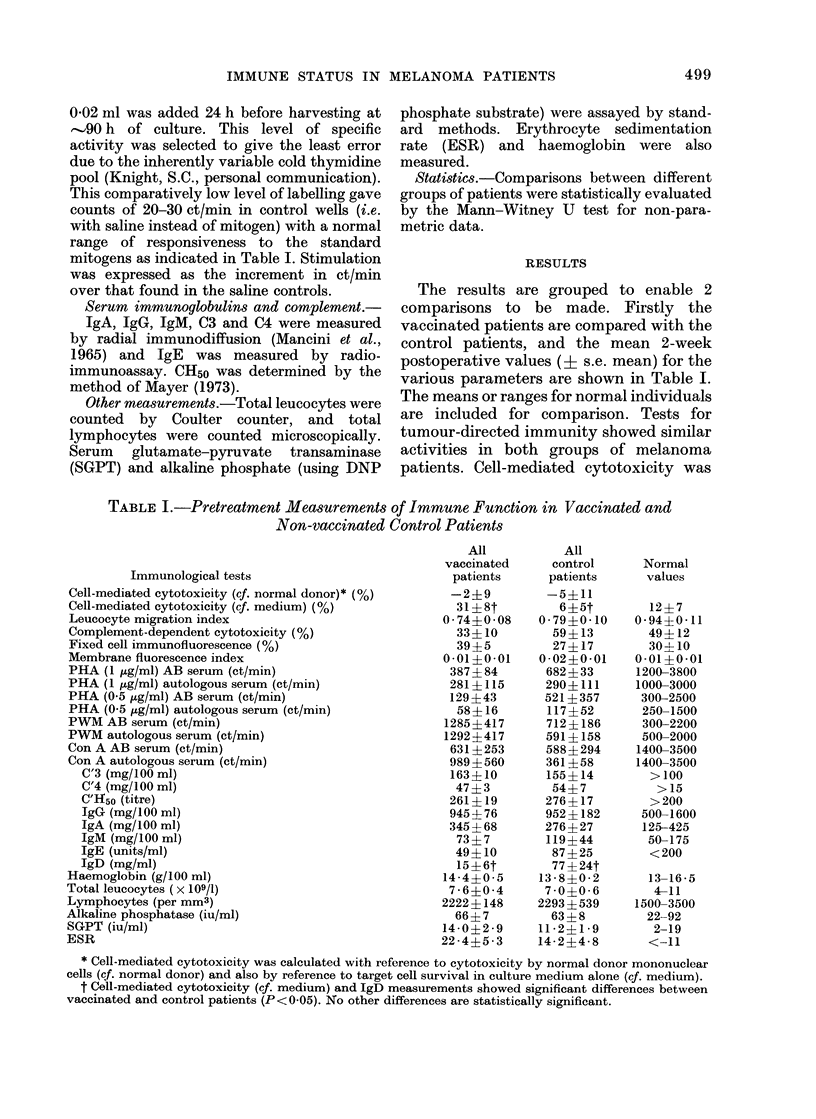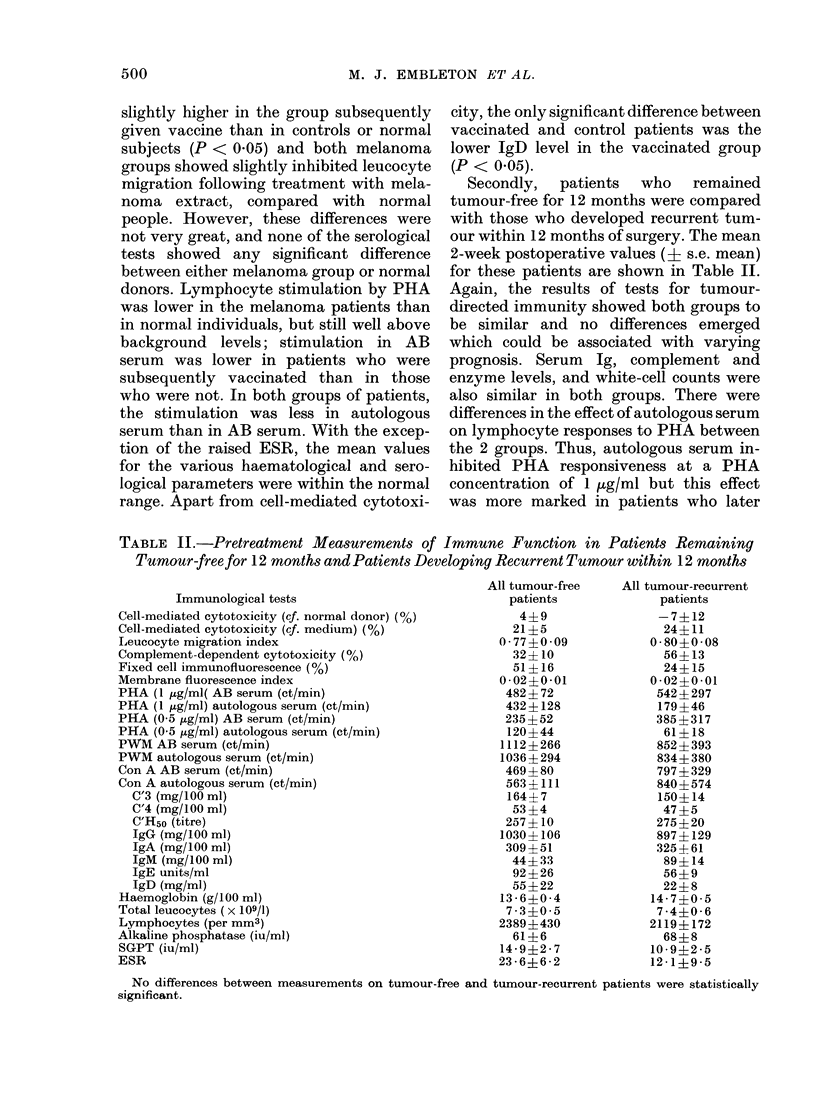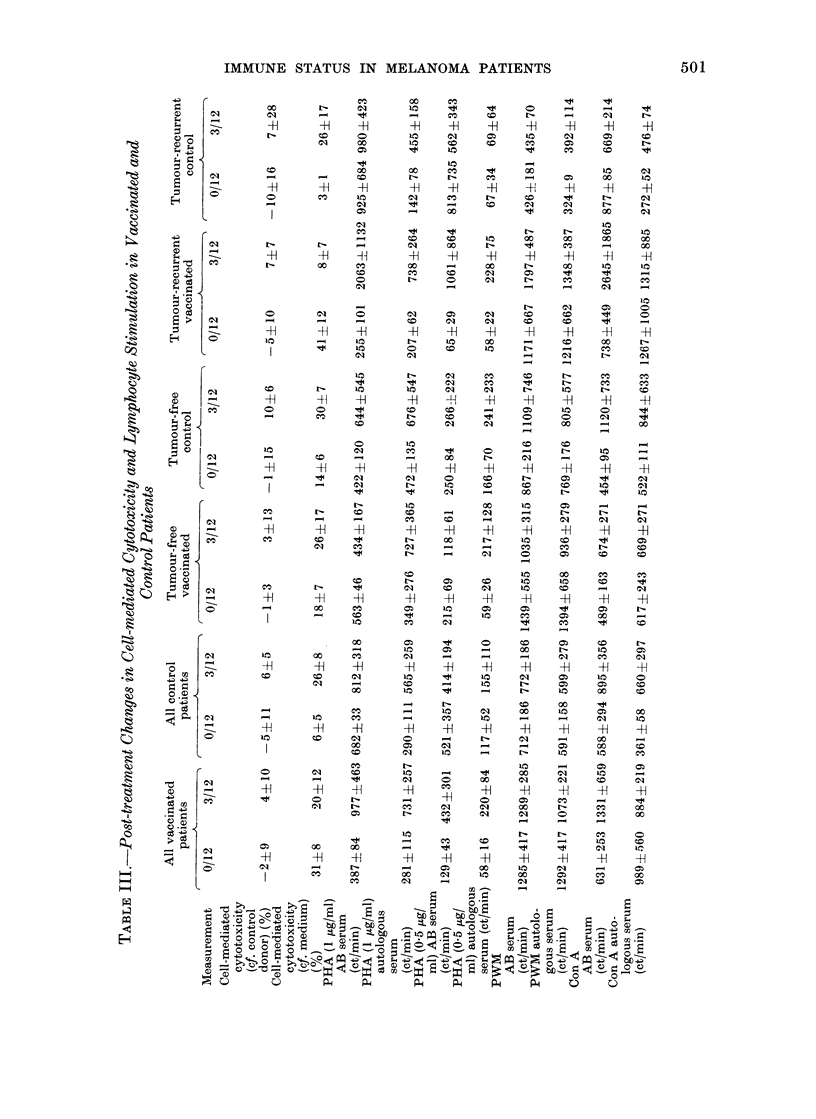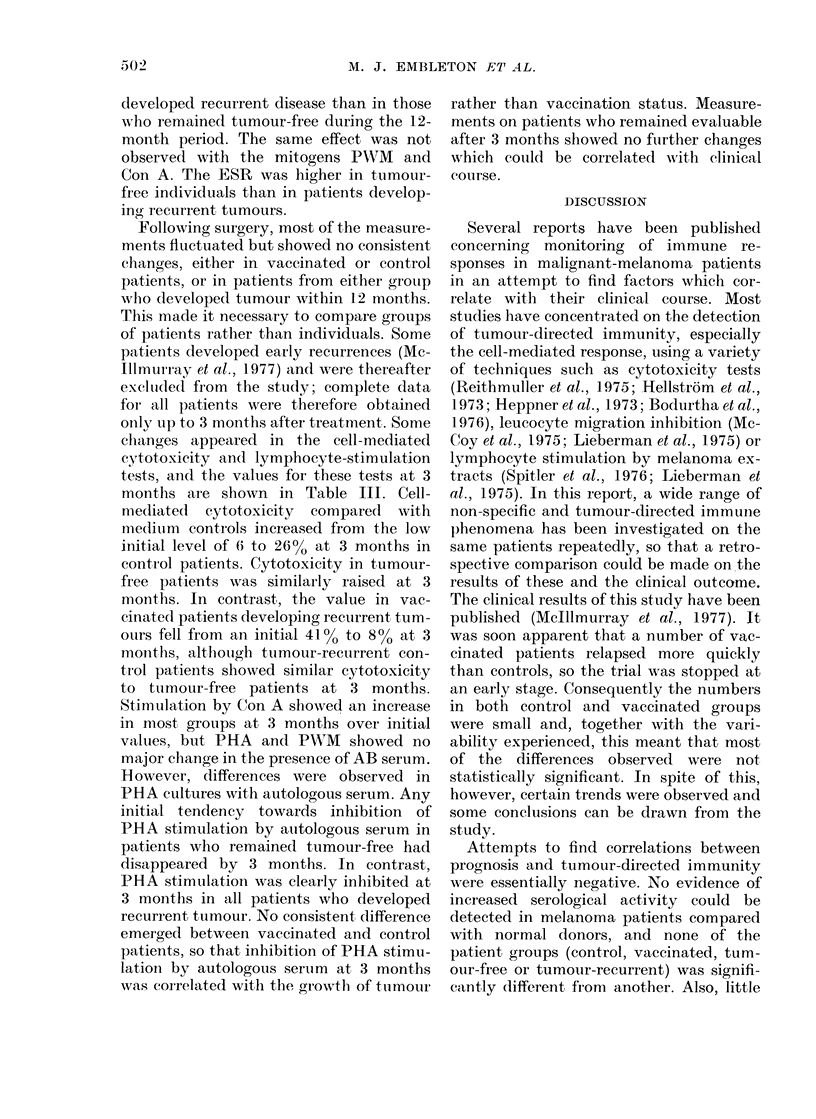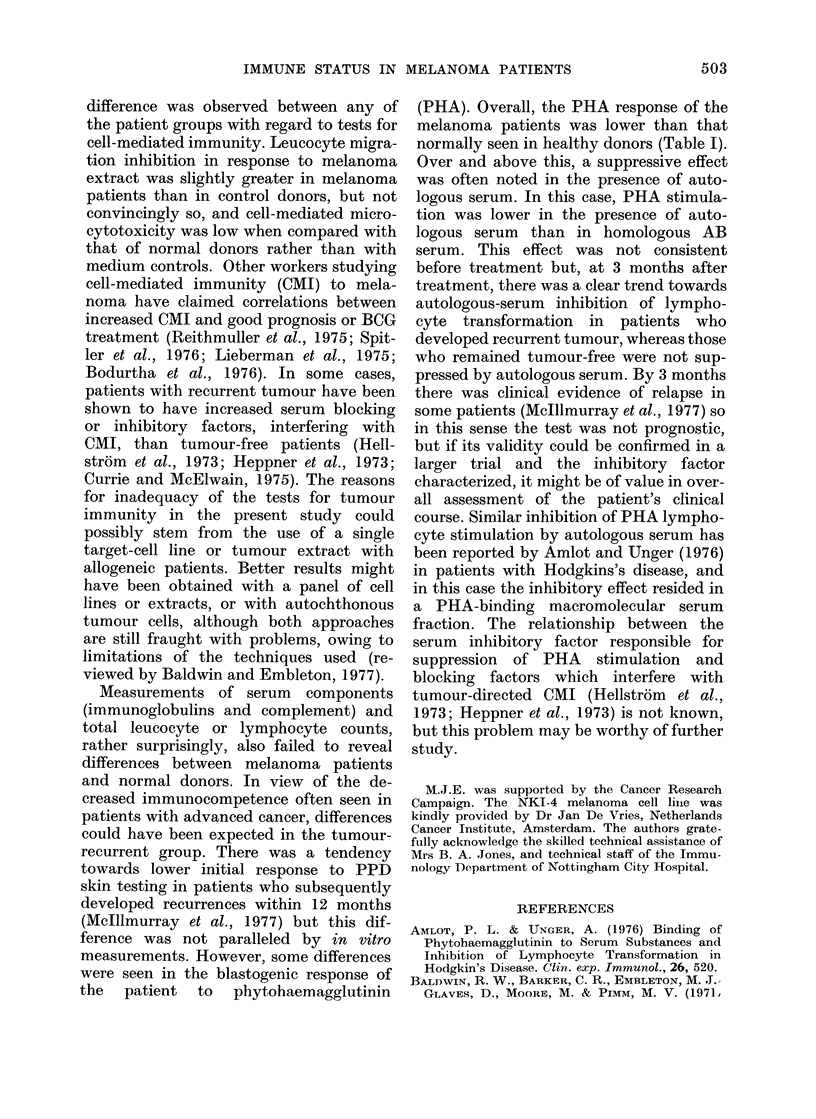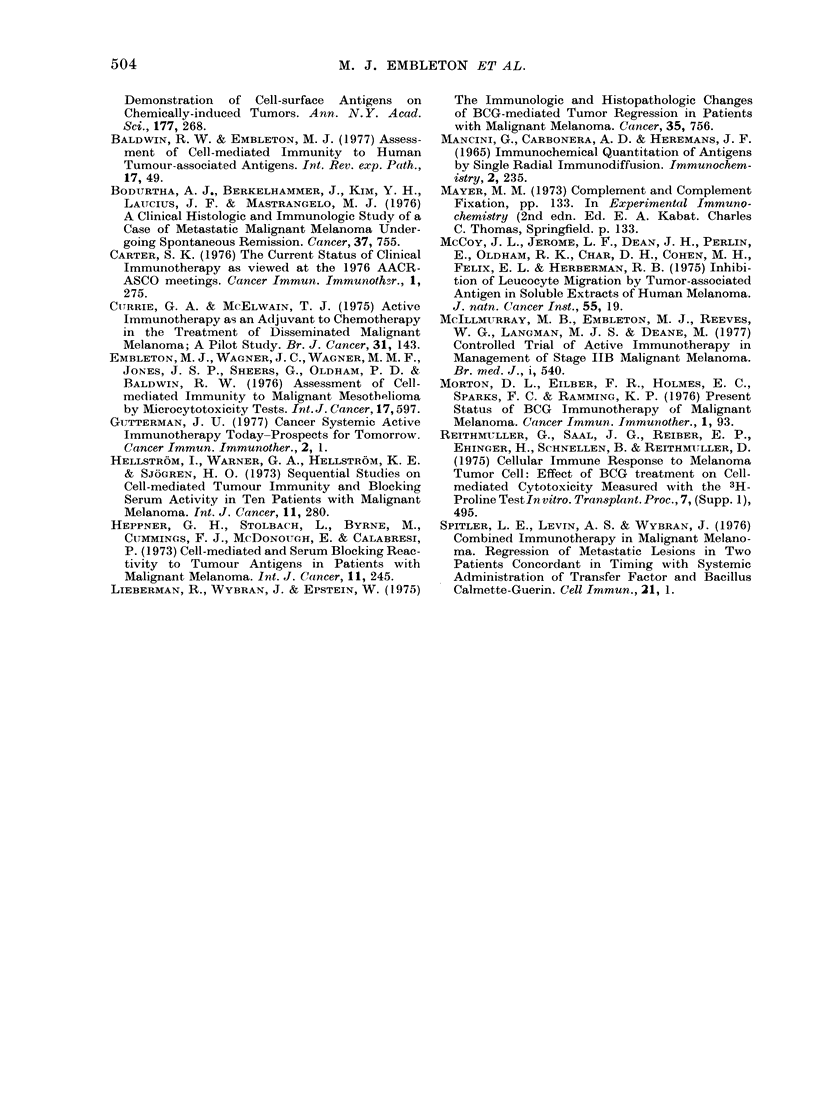# Immunological monitoring in a controlled trial of immunotherapy in stage IIB malignant melanoma.

**DOI:** 10.1038/bjc.1978.77

**Published:** 1978-04

**Authors:** M. J. Embleton, J. H. Ransom, M. B. McIllmurray, W. G. Reeves

## Abstract

Fifteen patients undergoing surgery for Stage IIb malignant melanoma were randomly allocated either to a group who received a vaccine of BCG mixed with irradiated autologous melanoma cells, or a control group who received no further treatment. All patients were monitored sequentially for immunological competence and tumour-directed immunity, using a wide range of techniques, and the results were compared retrospectively with their clinical course. Three months after surgery, there was a trend towards inhibition of PHA-induced lymphocyte transformation by autologous serum in patients who developed recurrent tumour within 12 months after treatment. Serum from patients who remained tumour-free for 12 months did not inhibit stimulation of autologous lymphocytes by PHA. Apart from this test, no other immunological parameters correlated either with clinical course or with the type of treatment received.


Br. J. Cancer (1978) 37, 497

### IMMUNOLOGICAL MONITORING IN A CONTROLLED TRIAL OF IMMUNOTHERAPY IN STAGE IIB MALIGNANT MELANOMA

M. J. EMBLETON,* J. H. RANSOM,† M. B. McILLMURRAY‡ AND W. G. REEVES†

*From the *Cancer Research Campaign Laboratory, †Department of Immunology, and ‡Department of Therapeutics, University of Nottingham, Nottingham*

Received 27 October 1977 Accepted 18 January 1978

**Summary.—Fifteen patients undergoing surgery for Stage IIb malignant melanoma were randomly allocated either to a group who received a vaccine of BCG mixed with irradiated autologous melanoma cells, or a control group who received no further treatment. All patients were monitored sequentially for immunological competence and tumour-directed immunity, using a wide range of techniques, and the results were compared retrospectively with their clinical course.**

**Three months after surgery, there was a trend towards inhibition of PHA-induced lymphocyte transformation by autologous serum in patients who developed recurrent tumour within 12 months after treatment. Serum from patients who remained tumour-free for 12 months did not inhibit stimulation of autologous lymphocytes by PHA. Apart from this test, no other immunological parameters correlated either with clinical course or with the type of treatment received.**

SEVERAL trials of immunotherapy in patients with malignant melanoma using bacterial adjuvants are currently in progress or have been reported (Carter, 1976; Gutterman, 1977; Morton *et al.*, 1976). In some of these trials, attempts have been made to monitor the immune status of patients with regard to tumour-directed immunity or general immunocompetence. In most reports only a limited number of different parameters have been measured.

We have recently published clinical results of a controlled trial of active immunotherapy in Stage IIb melanoma, using a vaccine of irradiated autologous melanoma cells mixed with BCG (McIllmurray *et al.*, 1977). Patients in the trial were systematically monitored throughout for levels of immunocompetence and evidence of tumour immunity, using a wide range of techniques, in order to identify changes which could be correlated with their clinical course, the primary objective being to determine ways of identifying patients who would benefit from immunotherapy. The skin response of these patients to the recall antigens PPD and Varidase has been reported (McIllmurray *et al.*, 1977), and the following reports their immune responses as measured by various *in vitro* tests.

### MATERIALS AND METHODS

*Patients.*—Fifteen patients with malignant melanoma were treated surgically for localized recurrent tumour following an original operation. They were judged to be tumour-free after thorough clinical examination and after isotope scans of liver, bone and brain, and radiographs of chest and skeleton were shown to be normal. They were then allocated at random to either a treatment group (8 patients) which received a vaccine postoperatively, or a control group (7 patients) without further treatment. The vaccine consisted of a mixture of live BCG (Glaxo, $3 \times 10^7$ organisms) and $5 \times 10^7$ autologous irradiated tumour cells, and was given intradermally on the 14th postoperative day as described previously (McIllmurray *et al.*, 1977). No additional treatment was given to the patients in either group during the period of study. Comparison of control and vaccinated patients showed that they were broadly comparable in age, site of primary tumour and extent of disease. Patients in both groups were monitored 2 weeks post-operatively and at intervals of 1, 2, 3, 6 and 12 months thereafter, during which time some of them developed further tumour recurrence.

*Cell-mediated cytotoxicity tests.*—Cell-mediated cytotoxicity was measured against a long-term melanoma cell line, NKl-4, using a previously described microcytotoxicity test (Embleton *et al.*, 1976). Target cells were plated in Cooke M29 ART plates at 200 per well, and after attachment $5 \times 10^4$ effector cells from patients or normal donors were added to 8 replicate wells. These effector cells were prepared from heparinized venous blood by centrifugation on Ficoll/Triosil. Different normal donors were used on separate occasions. After 2 days' incubation at 37°C the surviving target cells were stained and counted. Percentage cytotoxicity by melanoma patients' effector cells was calculated by comparison with effector cells from normal donors and with target-cell wells treated with culture medium alone.

*Leucocyte Migration inhibition.*—A 10,000 *g* supernatant extract was prepared from a homogenate of pooled melanoma tissue in phosphate-buffered saline (PBS, pH 7·2) and was stored in the vapour phase of a liquid $N_2$ bank. Leucocytes were prepared by sedimentation of heparinized blood for 30 min with 2 ml 3% gelatin per 20 ml blood. The leucocyte-rich supernatant was collected and the cells washed twice, and $\sim 10^7$ cells were incubated in 0·2 ml of PBS supplemented with melanoma extract at concentrations of 10, 100 and 500 μg. After 2 h incubation $\sim 2{\cdot}5 \times 10^6$ cells were introduced into triplicate 50 μl glass capillary tubes. The ends were sealed and the tubes centrifuged to produce a cell button. The tubes were cut at the cell/liquid interface and the stubs with the cell buttons were mounted in plastic plates (Sterilin) in Eagle's MEM plus 10% foetal calf serum. The leucocytes were allowed to migrate at 37°C for 18 h and the areas of migration were measured by planimetry. A migration index was calculated as:

$$\frac{\text{Migration area after antigen treatment}}{\text{Migration area after PBS treatment}}$$

*Complement-dependent cytotoxicity.*—NKl-4 melanoma cells ($5 \times 10^4$ in 100 μl MEM) were incubated with 100 μl test serum or AB control serum for 20 min at 37°C. Rabbit serum (300 μl 1/3 dilution in MEM) was added for a further 60 min incubation. Aliquots of 10 μl were plated in 6 wells of a Cooke microtitration plate and incubated 18 h at 37°C. Surviving cells were stained and counted, and cytotoxicity was calculated by comparison with the AB control serum.

*Immunofluorescence.*—Slides were prepared with 10 separate spots of $10^4$ NKl-4 cells. They were air-dried and fixed with acetone at −20°C. A drop of test or AB control serum diluted 1/5 was placed on a patch of fixed cells and incubated for 20 min at room temperature in a humidified chamber. The slide was washed ×3 and a drop of 1/40 dilution FITC-conjugated sheep anti-human Ig (Burroughs Wellcome) was added. After 20 min the slide was again washed ×3 and the cells were flooded with PBS:glycerol (1:1). The cells were examined under incident blue-light fluorescence and the percentage of cells showing cytoplasmic fluorescence was scored.

Viable cells in suspension were used in a membrane fluorescence test as previously described (Baldwin *et al.*, 1971). Aliquots of $5 \times 10^5$ NKl-4 cells were incubated with 50 μl test or AB control serum for 20 min at room temperature. After 3 washes the cells were incubated with 50 μl 1/20 FITC-conjugated sheep anti-human Ig for 20 min. The cells were again washed ×3 and suspended in 100 μl PBS:glycerol (1:1). They were examined under transmitted UV illumination and a fluorescence index was calculated as:

$$\frac{\%\text{ cells unstained in control} - \%\text{ cells unstained in test serum}}{\%\text{ cells unstained in control}}$$

*Lymphocyte-stimulation tests.*—Leucocytes were prepared from defibrinated venous blood by plasmagel sedimentation of red cells. Aliquots of $2 \times 10^5$ leucocytes were set up in culture in microtest plates using RPMI 1640 medium with a bicarbonate/$CO_2$ buffer system. The individual culture volumes of 0·22 ml included 10% autologous or AB serum. The following mitogens were added (or saline as control) in a further 0·02 ml: purified phytohaemagglutinin (Burroughs Wellcome; PHA) at 1·0 μg/ml and 0·5 μg/ml; concanavalin A (Miles: Con A) at 50 μg/ml, and pokeweed mitogen (Gibco; PWM) at 1/100 dilution. All concentrations given are the final concentration of the mitogen in the cultures.

$^3$H-thymidine (sp. act. 200 mCi/mmol) in

0·02 ml was added 24 h before harvesting at ~90 h of culture. This level of specific activity was selected to give the least error due to the inherently variable cold thymidine pool (Knight, S.C., personal communication). This comparatively low level of labelling gave counts of 20–30 ct/min in control wells (*i.e.* with saline instead of mitogen) with a normal range of responsiveness to the standard mitogens as indicated in Table I. Stimulation was expressed as the increment in ct/min over that found in the saline controls.

*Serum immunoglobulins and complement.*—IgA, IgG, IgM, C3 and C4 were measured by radial immunodiffusion (Mancini *et al.*, 1965) and IgE was measured by radioimmunoassay. $CH_{50}$ was determined by the method of Mayer (1973).

*Other measurements.*—Total leucocytes were counted by Coulter counter, and total lymphocytes were counted microscopically. Serum glutamate–pyruvate transaminase (SGPT) and alkaline phosphate (using DNP phosphate substrate) were assayed by standard methods. Erythrocyte sedimentation rate (ESR) and haemoglobin were also measured.

*Statistics.*—Comparisons between different groups of patients were statistically evaluated by the Mann–Witney U test for non-parametric data.

### RESULTS

The results are grouped to enable 2 comparisons to be made. Firstly the vaccinated patients are compared with the control patients, and the mean 2-week postoperative values (± s.e. mean) for the various parameters are shown in Table I. The means or ranges for normal individuals are included for comparison. Tests for tumour-directed immunity showed similar activities in both groups of melanoma patients. Cell-mediated cytotoxicity was

TABLE I.—*Pretreatment Measurements of Immune Function in Vaccinated and Non-vaccinated Control Patients*

| Immunological tests | All vaccinated patients | All control patients | Normal values |
|---|---|---|---|
| Cell-mediated cytotoxicity (*cf.* normal donor)* (%) | −2±9 | −5±11 | |
| Cell-mediated cytotoxicity (*cf.* medium) (%) | 31±8† | 6±5† | 12±7 |
| Leucocyte migration index | 0·74±0·08 | 0·79±0·10 | 0·94±0·11 |
| Complement-dependent cytotoxicity (%) | 33±10 | 59±13 | 49±12 |
| Fixed cell immunofluorescence (%) | 39±5 | 27±17 | 30±10 |
| Membrane fluorescence index | 0·01±0·01 | 0·02±0·01 | 0·01±0·01 |
| PHA (1 μg/ml) AB serum (ct/min) | 387±84 | 682±33 | 1200–3800 |
| PHA (1 μg/ml) autologous serum (ct/min) | 281±115 | 290±111 | 1000–3000 |
| PHA (0·5 μg/ml) AB serum (ct/min) | 129±43 | 521±357 | 300–2500 |
| PHA (0·5 μg/ml) autologous serum (ct/min) | 58±16 | 117±52 | 250–1500 |
| PWM AB serum (ct/min) | 1285±417 | 712±186 | 300–2200 |
| PWM autologous serum (ct/min) | 1292±417 | 591±158 | 500–2000 |
| Con A AB serum (ct/min) | 631±253 | 588±294 | 1400–3500 |
| Con A autologous serum (ct/min) | 989±560 | 361±58 | 1400–3500 |
| C′3 (mg/100 ml) | 163±10 | 155±14 | >100 |
| C′4 (mg/100 ml) | 47±3 | 54±7 | >15 |
| $C'H_{50}$ (titre) | 261±19 | 276±17 | >200 |
| IgG (mg/100 ml) | 945±76 | 952±182 | 500–1600 |
| IgA (mg/100 ml) | 345±68 | 276±27 | 125–425 |
| IgM (mg/100 ml) | 73±7 | 119±44 | 50–175 |
| IgE (units/ml) | 49±10 | 87±25 | <200 |
| IgD (mg/ml) | 15±6† | 77±24† | |
| Haemoglobin (g/100 ml) | 14·4±0·5 | 13·8±0·2 | 13–16·5 |
| Total leucocytes ($\times 10^9$/l) | 7·6±0·4 | 7·0±0·6 | 4–11 |
| Lymphocytes (per $mm^3$) | 2222±148 | 2293±539 | 1500–3500 |
| Alkaline phosphatase (iu/ml) | 66±7 | 63±8 | 22–92 |
| SGPT (iu/ml) | 14·0±2·9 | 11·2±1·9 | 2–19 |
| ESR | 22·4±5·3 | 14·2±4·8 | <–11 |

* Cell-mediated cytotoxicity was calculated with reference to cytotoxicity by normal donor mononuclear cells (*cf.* normal donor) and also by reference to target cell survival in culture medium alone (*cf.* medium).

† Cell-mediated cytotoxicity (*cf.* medium) and IgD measurements showed significant differences between vaccinated and control patients ($P<0·05$). No other differences are statistically significant.

slightly higher in the group subsequently given vaccine than in controls or normal subjects ($P < 0 \cdot 05$) and both melanoma groups showed slightly inhibited leucocyte migration following treatment with melanoma extract, compared with normal people. However, these differences were not very great, and none of the serological tests showed any significant difference between either melanoma group or normal donors. Lymphocyte stimulation by PHA was lower in the melanoma patients than in normal individuals, but still well above background levels; stimulation in AB serum was lower in patients who were subsequently vaccinated than in those who were not. In both groups of patients, the stimulation was less in autologous serum than in AB serum. With the exception of the raised ESR, the mean values for the various haematological and serological parameters were within the normal range. Apart from cell-mediated cytotoxicity, the only significant difference between vaccinated and control patients was the lower IgD level in the vaccinated group ($P < 0 \cdot 05$).

Secondly, patients who remained tumour-free for 12 months were compared with those who developed recurrent tumour within 12 months of surgery. The mean 2-week postoperative values ($\pm$ s.e. mean) for these patients are shown in Table II. Again, the results of tests for tumour-directed immunity showed both groups to be similar and no differences emerged which could be associated with varying prognosis. Serum Ig, complement and enzyme levels, and white-cell counts were also similar in both groups. There were differences in the effect of autologous serum on lymphocyte responses to PHA between the 2 groups. Thus, autologous serum inhibited PHA responsiveness at a PHA concentration of 1 $\mu$g/ml but this effect was more marked in patients who later

TABLE II.—*Pretreatment Measurements of Immune Function in Patients Remaining Tumour-free for* 12 *months and Patients Developing Recurrent Tumour within* 12 *months*

| Immunological tests | All tumour-free patients | All tumour-recurrent patients |
|---|---|---|
| Cell-mediated cytotoxicity (*cf.* normal donor) (%) | $4 \pm 9$ | $-7 \pm 12$ |
| Cell-mediated cytotoxicity (*cf.* medium) (%) | $21 \pm 5$ | $24 \pm 11$ |
| Leucocyte migration index | $0 \cdot 77 \pm 0 \cdot 09$ | $0 \cdot 80 \pm 0 \cdot 08$ |
| Complement-dependent cytotoxicity (%) | $32 \pm 10$ | $56 \pm 13$ |
| Fixed cell immunofluorescence (%) | $51 \pm 16$ | $24 \pm 15$ |
| Membrane fluorescence index | $0 \cdot 02 \pm 0 \cdot 01$ | $0 \cdot 02 \pm 0 \cdot 01$ |
| PHA (1 $\mu$g/ml( AB serum (ct/min) | $482 \pm 72$ | $542 \pm 297$ |
| PHA (1 $\mu$g/ml) autologous serum (ct/min) | $432 \pm 128$ | $179 \pm 46$ |
| PHA (0·5 $\mu$g/ml) AB serum (ct/min) | $235 \pm 52$ | $385 \pm 317$ |
| PHA (0·5 $\mu$g/ml) autologous serum (ct/min) | $120 \pm 44$ | $61 \pm 18$ |
| PWM AB serum (ct/min) | $1112 \pm 266$ | $852 \pm 393$ |
| PWM autologous serum (ct/min) | $1036 \pm 294$ | $834 \pm 380$ |
| Con A AB serum (ct/min) | $469 \pm 80$ | $797 \pm 329$ |
| Con A autologous serum (ct/min) | $563 \pm 111$ | $840 \pm 574$ |
| C'3 (mg/100 ml) | $164 \pm 7$ | $150 \pm 14$ |
| C'4 (mg/100 ml) | $53 \pm 4$ | $47 \pm 5$ |
| C'$H_{50}$ (titre) | $257 \pm 10$ | $275 \pm 20$ |
| IgG (mg/100 ml) | $1030 \pm 106$ | $897 \pm 129$ |
| IgA (mg/100 ml) | $309 \pm 51$ | $325 \pm 61$ |
| IgM (mg/100 ml) | $44 \pm 33$ | $89 \pm 14$ |
| IgE units/ml | $92 \pm 26$ | $56 \pm 9$ |
| IgD (mg/ml) | $55 \pm 22$ | $22 \pm 8$ |
| Haemoglobin (g/100 ml) | $13 \cdot 6 \pm 0 \cdot 4$ | $14 \cdot 7 \pm 0 \cdot 5$ |
| Total leucocytes ($\times 10^9$/l) | $7 \cdot 3 \pm 0 \cdot 5$ | $7 \cdot 4 \pm 0 \cdot 6$ |
| Lymphocytes (per $mm^3$) | $2389 \pm 430$ | $2119 \pm 172$ |
| Alkaline phosphatase (iu/ml) | $61 \pm 6$ | $68 \pm 8$ |
| SGPT (iu/ml) | $14 \cdot 9 \pm 2 \cdot 7$ | $10 \cdot 9 \pm 2 \cdot 5$ |
| ESR | $23 \cdot 6 \pm 6 \cdot 2$ | $12 \cdot 1 \pm 9 \cdot 5$ |

No differences between measurements on tumour-free and tumour-recurrent patients were statistically significant.

TABLE III.—*Post-treatment Changes in Cell-mediated Cytotoxicity and Lymphocyte Stimulation in Vaccinated and Control Patients*

| | All vaccinated patients | | All control patients | | Tumour-free vaccinated | | Tumour-free control | | Tumour-recurrent vaccinated | | Tumour-recurrent control | |
|---|---|---|---|---|---|---|---|---|---|---|---|---|
| Measurement | 0/12 | 3/12 | 0/12 | 3/12 | 0/12 | 3/12 | 0/12 | 3/12 | 0/12 | 3/12 | 0/12 | 3/12 |
| Cell-mediated cytotoxicity (*cf.* control donor) (%) | −2±9 | 4±10 | −5±11 | 6±5 | −1±3 | 3±13 | −1±15 | 10±6 | −5±10 | 7±7 | −10±16 | 7±28 |
| Cell-mediated cytotoxicity (*cf.* medium) (%) | 31±8 | 20±12 | 6±5 | 26±8 | 18±7 | 26±17 | 14±6 | 30±7 | 41±12 | 8±7 | 3±1 | 26±17 |
| PHA (1 μg/ml) AB serum (ct/min) | 387±84 | 977±463 | 682±33 | 812±318 | 563±46 | 434±167 | 422±120 | 644±545 | 255±101 | 2063±1132 | 925±684 | 980±423 |
| PHA (1 μg/ml) autologous serum (ct/min) | 281±115 | 731±257 | 290±111 | 565±259 | 349±276 | 727±365 | 472±135 | 676±547 | 207±62 | 738±264 | 142±78 | 455±158 |
| PHA (0·5 μg/ml) AB serum (ct/min) | 129±43 | 432±301 | 521±357 | 414±194 | 215±69 | 118±61 | 250±84 | 266±222 | 65±29 | 1061±864 | 813±735 | 562±343 |
| PHA (0·5 μg/ml) autologous serum (ct/min) | 58±16 | 220±84 | 117±52 | 155±110 | 59±26 | 217±128 | 166±70 | 241±233 | 58±22 | 228±75 | 67±34 | 69±64 |
| PWM AB serum (ct/min) | 1285±417 | 1289±285 | 712±186 | 772±186 | 1439±555 | 1035±315 | 867±216 | 1109±746 | 1171±667 | 1797±487 | 426±181 | 435±70 |
| PWM autologous serum (ct/min) | 1292±417 | 1073±221 | 591±158 | 599±279 | 1394±658 | 936±279 | 769±176 | 805±577 | 1216±662 | 1348±387 | 324±9 | 392±114 |
| Con A AB serum (ct/min) | 631±253 | 1331±659 | 588±294 | 895±356 | 489±163 | 674±271 | 454±95 | 1120±733 | 738±449 | 2645±1865 | 877±85 | 669±214 |
| Con A autologous serum (ct/min) | 989±560 | 884±219 | 361±58 | 660±297 | 617±243 | 669±271 | 522±111 | 844±633 | 1267±1005 | 1315±885 | 272±52 | 476±74 |

developed recurrent disease than in those who remained tumour-free during the 12-month period. The same effect was not observed with the mitogens PWM and Con A. The ESR was higher in tumour-free individuals than in patients developing recurrent tumours.

Following surgery, most of the measurements fluctuated but showed no consistent changes, either in vaccinated or control patients, or in patients from either group who developed tumour within 12 months. This made it necessary to compare groups of patients rather than individuals. Some patients developed early recurrences (McIllmurray *et al.*, 1977) and were thereafter excluded from the study; complete data for all patients were therefore obtained only up to 3 months after treatment. Some changes appeared in the cell-mediated cytotoxicity and lymphocyte-stimulation tests, and the values for these tests at 3 months are shown in Table III. Cell-mediated cytotoxicity compared with medium controls increased from the low initial level of 6 to 26% at 3 months in control patients. Cytotoxicity in tumour-free patients was similarly raised at 3 months. In contrast, the value in vaccinated patients developing recurrent tumours fell from an initial 41% to 8% at 3 months, although tumour-recurrent control patients showed similar cytotoxicity to tumour-free patients at 3 months. Stimulation by Con A showed an increase in most groups at 3 months over initial values, but PHA and PWM showed no major change in the presence of AB serum. However, differences were observed in PHA cultures with autologous serum. Any initial tendency towards inhibition of PHA stimulation by autologous serum in patients who remained tumour-free had disappeared by 3 months. In contrast, PHA stimulation was clearly inhibited at 3 months in all patients who developed recurrent tumour. No consistent difference emerged between vaccinated and control patients, so that inhibition of PHA stimulation by autologous serum at 3 months was correlated with the growth of tumour rather than vaccination status. Measurements on patients who remained evaluable after 3 months showed no further changes which could be correlated with clinical course.

### DISCUSSION

Several reports have been published concerning monitoring of immune responses in malignant-melanoma patients in an attempt to find factors which correlate with their clinical course. Most studies have concentrated on the detection of tumour-directed immunity, especially the cell-mediated response, using a variety of techniques such as cytotoxicity tests (Reithmuller *et al.*, 1975; Hellström *et al.*, 1973; Heppner *et al.*, 1973; Bodurtha *et al.*, 1976), leucocyte migration inhibition (McCoy *et al.*, 1975; Lieberman *et al.*, 1975) or lymphocyte stimulation by melanoma extracts (Spitler *et al.*, 1976; Lieberman *et al.*, 1975). In this report, a wide range of non-specific and tumour-directed immune phenomena has been investigated on the same patients repeatedly, so that a retrospective comparison could be made on the results of these and the clinical outcome. The clinical results of this study have been published (McIllmurray *et al.*, 1977). It was soon apparent that a number of vaccinated patients relapsed more quickly than controls, so the trial was stopped at an early stage. Consequently the numbers in both control and vaccinated groups were small and, together with the variability experienced, this meant that most of the differences observed were not statistically significant. In spite of this, however, certain trends were observed and some conclusions can be drawn from the study.

Attempts to find correlations between prognosis and tumour-directed immunity were essentially negative. No evidence of increased serological activity could be detected in melanoma patients compared with normal donors, and none of the patient groups (control, vaccinated, tumour-free or tumour-recurrent) was significantly different from another. Also, little difference was observed between any of the patient groups with regard to tests for cell-mediated immunity. Leucocyte migration inhibition in response to melanoma extract was slightly greater in melanoma patients than in control donors, but not convincingly so, and cell-mediated microcytotoxicity was low when compared with that of normal donors rather than with medium controls. Other workers studying cell-mediated immunity (CMI) to melanoma have claimed correlations between increased CMI and good prognosis or BCG treatment (Reithmuller *et al.*, 1975; Spitler *et al.*, 1976; Lieberman *et al.*, 1975; Bodurtha *et al.*, 1976). In some cases, patients with recurrent tumour have been shown to have increased serum blocking or inhibitory factors, interfering with CMI, than tumour-free patients (Hellström *et al.*, 1973; Heppner *et al.*, 1973; Currie and McElwain, 1975). The reasons for inadequacy of the tests for tumour immunity in the present study could possibly stem from the use of a single target-cell line or tumour extract with allogeneic patients. Better results might have been obtained with a panel of cell lines or extracts, or with autochthonous tumour cells, although both approaches are still fraught with problems, owing to limitations of the techniques used (reviewed by Baldwin and Embleton, 1977).

Measurements of serum components (immunoglobulins and complement) and total leucocyte or lymphocyte counts, rather surprisingly, also failed to reveal differences between melanoma patients and normal donors. In view of the decreased immunocompetence often seen in patients with advanced cancer, differences could have been expected in the tumour-recurrent group. There was a tendency towards lower initial response to PPD skin testing in patients who subsequently developed recurrences within 12 months (McIllmurray *et al.*, 1977) but this difference was not paralleled by *in vitro* measurements. However, some differences were seen in the blastogenic response of the patient to phytohaemagglutinin (PHA). Overall, the PHA response of the melanoma patients was lower than that normally seen in healthy donors (Table I). Over and above this, a suppressive effect was often noted in the presence of autologous serum. In this case, PHA stimulation was lower in the presence of autologous serum than in homologous AB serum. This effect was not consistent before treatment but, at 3 months after treatment, there was a clear trend towards autologous-serum inhibition of lymphocyte transformation in patients who developed recurrent tumour, whereas those who remained tumour-free were not suppressed by autologous serum. By 3 months there was clinical evidence of relapse in some patients (McIllmurray *et al.*, 1977) so in this sense the test was not prognostic, but if its validity could be confirmed in a larger trial and the inhibitory factor characterized, it might be of value in overall assessment of the patient's clinical course. Similar inhibition of PHA lymphocyte stimulation by autologous serum has been reported by Amlot and Unger (1976) in patients with Hodgkins's disease, and in this case the inhibitory effect resided in a PHA-binding macromolecular serum fraction. The relationship between the serum inhibitory factor responsible for suppression of PHA stimulation and blocking factors which interfere with tumour-directed CMI (Hellström *et al.*, 1973; Heppner *et al.*, 1973) is not known, but this problem may be worthy of further study.

M.J.E. was supported by the Cancer Research Campaign. The NKI-4 melanoma cell line was kindly provided by Dr Jan De Vries, Netherlands Cancer Institute, Amsterdam. The authors gratefully acknowledge the skilled technical assistance of Mrs B. A. Jones, and technical staff of the Immunology Department of Nottingham City Hospital.

### REFERENCES

AMLOT, P. L. & UNGER, A. (1976) Binding of Phytohaemagglutinin to Serum Substances and Inhibition of Lymphocyte Transformation in Hodgkin's Disease. *Clin. exp. Immunol.*, **26**, 520.

BALDWIN, R. W., BARKER, C. R., EMBLETON, M. J., GLAVES, D., MOORE, M. & PIMM, M. V. (1971)

Demonstration of Cell-surface Antigens on Chemically-induced Tumors. *Ann. N.Y. Acad. Sci.*, **177**, 268.

BALDWIN, R. W. & EMBLETON, M. J. (1977) Assessment of Cell-mediated Immunity to Human Tumour-associated Antigens. *Int. Rev. exp. Path.*, **17**, 49.

BODURTHA, A. J., BERKELHAMMER, J., KIM, Y. H., LAUCIUS, J. F. & MASTRANGELO, M. J. (1976) A Clinical Histologic and Immunologic Study of a Case of Metastatic Malignant Melanoma Undergoing Spontaneous Remission. *Cancer*, **37**, 755.

CARTER, S. K. (1976) The Current Status of Clinical Immunotherapy as viewed at the 1976 AACR-ASCO meetings. *Cancer Immun. Immunother.*, **1**, 275.

CURRIE, G. A. & McELWAIN, T. J. (1975) Active Immunotherapy as an Adjuvant to Chemotherapy in the Treatment of Disseminated Malignant Melanoma; A Pilot Study. *Br. J. Cancer*, **31**, 143.

EMBLETON, M. J., WAGNER, J. C., WAGNER, M. M. F., JONES, J. S. P., SHEERS, G., OLDHAM, P. D. & BALDWIN, R. W. (1976) Assessment of Cell-mediated Immunity to Malignant Mesothelioma by Microcytotoxicity Tests. *Int. J. Cancer*, **17**, 597.

GUTTERMAN, J. U. (1977) Cancer Systemic Active Immunotherapy Today–Prospects for Tomorrow. *Cancer Immun. Immunother.*, **2**, 1.

HELLSTRÖM, I., WARNER, G. A., HELLSTRÖM, K. E. & SJÖGREN, H. O. (1973) Sequential Studies on Cell-mediated Tumour Immunity and Blocking Serum Activity in Ten Patients with Malignant Melanoma. *Int. J. Cancer*, **11**, 280.

HEPPNER, G. H., STOLBACH, L., BYRNE, M., CUMMINGS, F. J., McDONOUGH, E. & CALABRESI, P. (1973) Cell-mediated and Serum Blocking Reactivity to Tumour Antigens in Patients with Malignant Melanoma. *Int. J. Cancer*, **11**, 245.

LIEBERMAN, R., WYBRAN, J. & EPSTEIN, W. (1975) The Immunologic and Histopathologic Changes of BCG-mediated Tumor Regression in Patients with Malignant Melanoma. *Cancer*, **35**, 756.

MANCINI, G., CARBONERA, A. D. & HEREMANS, J. F. (1965) Immunochemical Quantitation of Antigens by Single Radial Immunodiffusion. *Immunochemistry*, **2**, 235.

MAYER, M. M. (1973) Complement and Complement Fixation, pp. 133. In *Experimental Immunochemistry* (2nd edn. Ed. E. A. Kabat. Charles C. Thomas, Springfield. p. 133.

McCOY, J. L., JEROME, L. F., DEAN, J. H., PERLIN, E., OLDHAM, R. K., CHAR, D. H., COHEN, M. H., FELIX, E. L. & HERBERMAN, R. B. (1975) Inhibition of Leucocyte Migration by Tumor-associated Antigen in Soluble Extracts of Human Melanoma. *J. natn. Cancer Inst.*, **55**, 19.

McILLMURRAY, M. B., EMBLETON, M. J., REEVES, W. G., LANGMAN, M. J. S. & DEANE, M. (1977) Controlled Trial of Active Immunotherapy in Management of Stage IIB Malignant Melanoma. *Br. med. J.*, i, 540.

MORTON, D. L., EILBER, F. R., HOLMES, E. C., SPARKS, F. C. & RAMMING, K. P. (1976) Present Status of BCG Immunotherapy of Malignant Melanoma. *Cancer Immun. Immunother.*, **1**, 93.

REITHMULLER, G., SAAL, J. G., REIBER, E. P., EHINGER, H., SCHNELLEN, B. & REITHMULLER, D. (1975) Cellular Immune Response to Melanoma Tumor Cell: Effect of BCG treatment on Cell-mediated Cytotoxicity Measured with the $^3$H-Proline Test *In vitro*. *Transplant. Proc.*, **7**, (Supp. 1), 495.

SPITLER, L. E., LEVIN, A. S. & WYBRAN, J. (1976) Combined Immunotherapy in Malignant Melanoma. Regression of Metastatic Lesions in Two Patients Concordant in Timing with Systemic Administration of Transfer Factor and Bacillus Calmette-Guerin. *Cell Immun.*, **21**, 1.